# Genome editing between wonder and rejection

**DOI:** 10.3389/fbioe.2026.1832039

**Published:** 2026-06-01

**Authors:** María Florencia Goberna, Evelina Gisela Lezcano, Andrés Castro Alegría, Eva Nara Pereira, Silverio Andrés Quintana, Andrea Alejandra Arrúa, Danilo Fernández Ríos

**Affiliations:** 1 Coordination of Innovation and Biotechnology, National Bioeconomy Directorate, Sub-secretariat of Agricultural Production and Forestry, Secretariat of Agriculture, Livestock and Fisheries, Buenos Aires, Argentina; 2 Doctorado en Ciencias Jurídicas, Universidad Iberoamericana, Asunción, Paraguay; 3 Dirección de Postgrado, Universidad San Carlos, Asunción, Paraguay; 4 Departamento de Biología Molecular y Biotecnología, Instituto de Investigaciones en Ciencias de la Salud, Universidad Nacional de Asunción, San Lorenzo, Paraguay; 5 Grupo de Investigación Mycology Investigation and Safety Team, Centro Multidisciplinario de Investigaciones Tecnológicas, Universidad Nacional de Asunción, San Lorenzo, Paraguay; 6 Grupo de Investigación en Ciencias Regulatorias y Bioinnovación, Facultad de Ciencias Exactas y Naturales, Universidad Nacional de Asunción, San Lorenzo, Paraguay; 7 Doctorado en Ciencias de la Educación, Facultad de Ciencias de la Educación y la Comunicación, Universidad Autónoma de Asunción, Asunción, Paraguay

**Keywords:** CRISPR-cas (clustered regularly interspaced short palindromic repeats-CRISPR associated) system, ethical conflicts, genome edited (GenEd), new breeding techniques (NBTs), regulatory framework and governance

## 1 Introduction

Recent advances in genome editing (GEd)^1^ have revolutionized modern biotechnology, opening new possibilities in medicine, agriculture, and biomedical research fields. However, this progress has raised ethical and social concerns. This article examines these controversies as they manifest across different domains of application and discusses how GEd can be aligned with the principles of justice, safety, sustainability, and social legitimacy through a transparent and coherent normative approach.

## 2 Current regulations?

**FIGURE 1:**
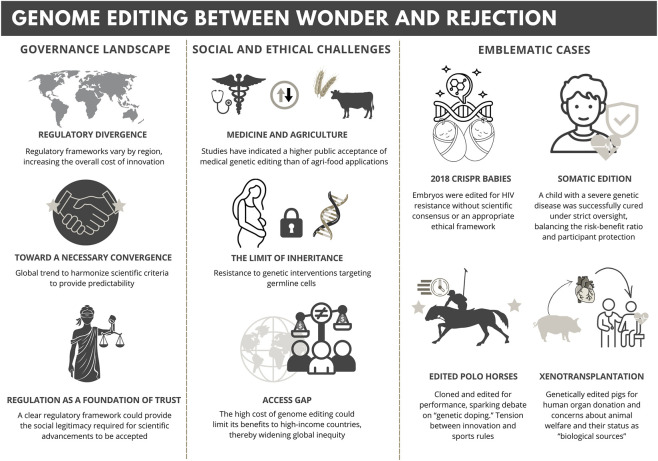
Genome-editing governance landscape. The conceptual figure provides a structured overview of the regulatory landscape, social-ethical challenges, and emblematic cases discussed in this article. This illustrates the divergence in regulations and efforts toward convergence, as well as the varying levels of public acceptance between medical and agricultural applications, including resistance to germline intervention and concerns about access and inequalities. Additionally, it highlights emblematic cases that have shaped public debate, such as the 2018 CRISPR babies, somatic gene therapy, edited polo horses, and xenotransplantation. Collectively, these themes reflect the dynamic tension between societal “wonder” and “rejection” that characterizes contemporary discussions on genome editing. The figure was created using the Canva Visual Suite. https://www.canva.com/visual-suite/.

Genome editing enables precise modifications of the genetic characteristics of organisms, facilitating improvements in productivity, resistance to biotic and abiotic stress, and animal health (Li et al., 2021; Kolluri et al., 2026; Zhao et al., 2026). However, its adoption still faces significant challenges due to the diversity of regulatory approaches across regions (Fernández Ríos et al., 2025b). The rules that govern these technologies vary considerably, and discussions regarding their scope often differ depending on the field of application. Ethical tensions surrounding GEd tend to become particularly visible in contexts where scientific innovation is closely connected to public policy, economic interests, and societal expectations, especially when comparing different areas, such as agriculture and biomedicine. In both contexts, the lack of clear regulatory criteria makes it more difficult to maintain the predictability and coherence of normative approaches. In many jurisdictions, regulatory triggers are associated with the use of genetic engineering. However, several countries have adopted product-based approaches^2^ in which organisms that do not contain a novel combination^3^ are comparable to those obtained through conventional breeding and are not regulated as genetically modified organisms (GMOs) (Fernández Ríos et al., 2025a; Mewett et al., 2026). Although technical in nature, these limitations can acquire ethical relevance when they affect the fairness, legitimacy, and consistency of public decision-making processes (Figure 1).

Recent political developments suggest an emerging trend toward partial regulatory convergence. In this context, the European Union is nearing the completion of a specific framework for plants developed using GEd (Ricroch et al., 2026). While some products resulting from this technology are treated as equivalent to outcomes that could arise naturally or through conventional breeding, others remain subject to additional requirements similar to those for GMOs (European Commission, 2025). This update may bring the European Union closer to science-based regulatory frameworks already adopted in countries such as Canada and the United States, as well as in several countries in Latin America, Africa, and Asia (Fernández Ríos et al., 2025b). These differences reflect distinct regulatory traditions and approaches to addressing uncertainty. In the European Union, GEd has been governed through a more precaution-oriented approach (Aerni, 2019), with a greater emphasis on managing uncertainty before broader regulatory acceptance. Other jurisdictions have adopted more product-based or case-by-case approaches, placing greater emphasis on proportionality, familiarity, and characteristics of the final product (Casacuberta and Puigdomènech, 2018; Gould et al., 2022). Consequently, regulatory divergence reflects different policy priorities regarding innovation, precaution, and equity.

Despite these recent advances, key issues remain unresolved, including labelling requirements, transparency of product-related information, and implications for international trade, particularly for products subject to more stringent oversight (Council of the European Union, 2025). These elements may lead to different approval timelines across jurisdictions, affecting the cost of development, innovation dynamics, and the ability of small and medium enterprises or public institutions to access markets. Although several GEd products have reached the commercial stage (Waltz, 2022; Grinstein, 2023), their availability may be limited by international trade challenges.^4^

In biotechnology, these differences reflect the ongoing challenge of balancing safety and social legitimacy while maintaining clear and coherent regulations. Advancing toward mechanisms of regulatory convergence^5^ and international cooperation has become a priority for governments, whether through the mutual recognition of product regulatory status (Hernández-Soto and Gatica-Arias, 2024), alignment of definitions, or adoption of product-based approaches. Regulation enables both the government and various public groups to engage with biotechnology. It also facilitates innovation, making its application socially acceptable, transparent, and legitimate. As part of a broader social system, regulatory systems influence agents’ behavior to ensure that they align with societal expectations (Ivanov et al., 2025).

## 3 Social perceptions and ethical asymmetries

Public perception of GEd ranges from enthusiasm for its potential benefits to rejection motivated by perceived risks. Responses are not shaped exclusively by concerns such as safety, regulation, or market control; they also reflect more deeply rooted intuitions about what is perceived as natural, acceptable, or appropriate forms of human intervention in living systems (Kuzma, 2023; Buddle et al., 2025; Pittman et al., 2026). In this sense, it is useful to distinguish between moral and ethical behavior. Morality refers to the set of intuitions, beliefs, and value judgments held by individuals or social groups, which are often influenced by cultural and emotional factors. Ethics involves a more explicit and structured reflection through which these judgments are examined and discussed in public and institutional settings (Bryant and Baggott la Velle, 2019c).

Some reactions to GEd may arise from intuitive responses to interventions perceived as crossing certain boundaries, sometimes described as reactions of “repugnance” toward what is seen as a violation of natural or moral limits (Kass, 1997). For instance, psychological essentialism, the belief that organisms possess an inherent and unchanging essence, can lead to resistance toward genetic interventions, as alterations are perceived to affect the identity of the organism (Benítez Candia et al., 2021). In this sense, perceptions that the natural world follows an intrinsic order that should not be altered may contribute to concerns about “unnaturalness” or the idea of “playing God.”

In addition, emotional responses such as “disgust,” thought to have evolved as mechanisms to avoid potential threats, may intensify negative reactions to certain applications, particularly in the context of food (Curtis et al., 2011; Tybur et al., 2013). Recognizing this helps explain why public attitudes are not always aligned with scientific assessments of risk and benefit and why certain applications may trigger strong responses, even when no clear harm has been demonstrated (Cámara et al., 2018; Scheufele and Krause, 2019; Dawson et al., 2022; Kuzma, 2023).

Differences in public perception are evident when comparing agrifood and biomedical applications of GEd. In the biomedical domain, empirical studies have shown that social acceptance varies depending on the type of application: higher acceptance is typically associated with therapeutic uses, while heritable interventions continue to raise significant ethical concerns (Scheufele and Krause, 2019). For instance, germline GEd,^6^ which involves interventions in oocytes, sperm, or embryos (Lezcano et al., 2025), remains one of the most persistently debated issues and is often met with greater resistance (Singh, 2021). Even in the case of therapeutic applications, acceptance is not unconditional (Harris, 2011). Adverse outcomes reported in gene therapy trials (Henderson et al., 2024; Kaiser, 2024; Puig-Serra et al., 2025) appear to influence how these applications are perceived, reinforcing uncertainty and increasing ethical scrutiny.^7^ However, these events occur within research processes involving multiple phases of clinical evaluation and under regulatory frameworks designed to assess and manage such risks.

A central distinction underlying these differences lies between therapeutic and enhancement-oriented applications. Therapeutic uses are generally understood as those aimed at preventing, treating, or alleviating serious diseases and are therefore more readily associated with medical necessity and moral justification. In contrast, enhancement applications seek to modify or optimize traits beyond what is considered clinically necessary, raising concerns about social pressure, inequality, and the normalization of interventions targeting non-pathological characteristics (Harris, 2011). Some scholars consider the elimination of serious hereditary diseases an expression of responsibility toward future generations, whereas others warn that expanding permissible uses may normalize interventions beyond therapeutic purposes (Pennings, 2020; Johnson and Bowman, 2022). Consequently, this debate requires distinguishing between therapeutic and enhancement applications and defining the corresponding regulatory criteria and mechanisms (Ishii, 2017; Schleidgen et al., 2020).

Moreover, advances in biomedical applications of GEd are often associated with high costs, complex infrastructure, and highly specialized clinical settings. Consequently, access is generally concentrated in high-income countries and among socioeconomically privileged groups, widening the gap between those who can benefit from cutting-edge treatments and those who remain excluded (WHO, 2021a). This raises ethical concerns regarding the balance between technological progress and distributive justice, in which exclusion translates into the inability to access potentially life-saving interventions. If such interventions are accessible only to a segment of the global population, GEd risks deepening inequalities rather than reducing them (Barlevy et al., 2024).

However, agricultural innovations are more frequently debated in terms of health and environmental risks, regulatory frameworks, and market implications (Herring and Paarlberg, 2016). This is compounded by structural distrust toward actors that have historically led agricultural development (Benítez Candia et al., 2021). Public responses to GEd in agri-food systems are frequently related to food safety, sustainability, perceived risks, uncertainty, and moral considerations (Nguyen et al., 2023). Acceptance does not necessarily increase when GEd is framed around concrete social benefits, suggesting that perceived utility is not the main driver of support (Busch et al., 2022; Gao et al., 2024). Risk perception is a source of resistance, especially when GEd is cognitively associated with older GMO debates or fears of unintended consequences (Atimango et al., 2024). These concerns are often framed not only in terms of measurable risks, but also through broader questions about human intervention in food systems and the perceived integrity of natural processes. These differences between biomedical and agrifood applications reflect not only the characteristics of the technologies themselves but also their intended purposes and broader context of use (Kuzma, 2023).

## 4 Balancing precaution and innovation

How can scientific evidence be integrated with cultural values and social expectations without undermining public legitimacy or obstructing socially valuable innovations? Previous studies have pointed toward governance models that acknowledge the dynamic nature of scientific knowledge and allow regulatory criteria to be revised in light of emerging evidence (Scheinerman and Sherkow, 2021; Asquer and Morrison, 2022). This adaptive governance approach (Vengadesen et al., 2025) should not be reduced to regulatory flexibility alone, but must also reflect broader principles in decision-making, including legitimacy, transparency, consistency, and proportionality. Excessive precaution may hinder innovations with the potential to deliver significant benefits, whereas policy frameworks that are not fit for purpose (Giddings, 2024; Lubieniechi et al., 2025a) may allow actors to operate outside effective institutional oversight, thereby undermining public trust. In this sense, uncertainty should neither be treated as a reason for paralysis nor ignored in the design of governance frameworks, but rather understood as a condition that requires adaptive responses capable of incorporating new evidence over time (Asquer and Morrison, 2022).

In this context, governance should be operationalized through mechanisms that reflect the diversity of GEd uses and their specific contexts of use. Rather than applying uniform schemes, proportional oversight mechanisms should be incorporated based on the characteristics and intended use of each case (Gould et al., 2022), supported by clear and transparent criteria guiding regulatory decision-making. This includes considering issues of access to and distribution of benefits as well as adapting evaluation criteria to the specific context of each intervention, whether related to human health, environmental release, or potential intergenerational effects. Regulatory processes should remain consistent and predictable across comparable cases, while retaining the flexibility needed to adapt to new developments. In this sense, legitimacy does not derive from direct public participation in every decision, but from the robustness, coherence, and reliability of institutions, as well as from the consistent application of criteria in the evaluation of risks and benefits.

Emblematic cases have exposed both the potential of GEd and the governance challenges arising from rapidly evolving technologies. In 2018, a researcher edited the genomes of human embryos to confer resistance to HIV, resulting in the birth of “CRISPR babies” (Normile, 2021; Wang et al., 2023). The procedure was performed without the approval of a research ethics committee or an adequate oversight. This case exposed the limitations of existing oversight arrangements and the absence of robust international governance mechanisms for human germline GEd. Organizations such as the World Health Organization (WHO, 2021a; 2021b), United Nations Educational, Scientific and Cultural Organization (Amelan, 2015), and scientific academies from multiple countries have advanced recommendations and international cooperation frameworks to prevent the recurrence of similar cases in the future.

In contrast, in May 2025, a successful clinical case was reported in which a child with a severe genetic disease was treated and cured using CRISPR-Cas-based therapy conducted in a regulated clinical trial under strict bioethical oversight (Ledford, 2025). This milestone demonstrates that when applied under rigorous protocols and appropriate supervision (Bloomfield et al., 2026), GEd can create new opportunities for precision medicine.

Xenotransplantation based on GEd animal organs designed to be compatible with the human immune system is a case that lies at the intersection of medicine and animal biotechnology. Interest has grown in response to the global shortage of organs available for transplantation. Thousands of patients die annually while awaiting donors. Genome editing offers the possibility of producing “on-demand” organs through pigs engineered to reduce rejection, improve compatibility, and enhance clinical safety (Cooper, 2023; Cooper et al., 2025). This raises ethical questions regarding the use of animals as “biological sources.” Xenotransplantation illustrates how transformative potential coexists with concerns related to animal welfare and social legitimacy. Even when biotechnology offers critically needed medical solutions, the balance between benefits, risks, and ethical responsibilities remains tenuous.

In a recent instance involving edited polo horses in Argentina (Miller, 2025), editing and cloning techniques were used to enhance traits associated with physical performance and endurance (Moro et al., 2020). This case ignited an international debate on the concept of “genetic doping” in equestrian sports, highlighting the tensions between biotechnological innovation and traditional sporting rules (Rosengren, 2022).

## 5 Discussion

Ethical debates surrounding GEd extend beyond traditional discussions about gene patenting (Bryant and Baggott la Velle, 2019b; Scheinerman and Sherkow, 2021), commercialization practices (Bryant and Baggott la Velle, 2019a), and limited access in low- and middle-income countries (Oluwole et al., 2023; Khaled et al., 2024). In this sense, regulation defines the boundaries of biotechnological advancements, identifies the stakeholders involved, the values that guide them, and the mechanisms that confer legitimacy.

The notion of “waiting for more evidence” can be as problematic as advancing without adequate controls, as both positions reveal asymmetric relationships with scientific uncertainty. These asymmetries are also geographical, as different regulatory traditions reflect different tolerances for uncertainty and different ways of balancing innovation, precaution and equity. They are also reflected across domains of application, as biomedical and agrifood uses of GEd tend to be evaluated through different ethical lenses and societal expectations. Governance models that allow criteria to be updated as new evidence becomes available could be an alternative approach (Vengadesen et al., 2025).

While continuously evolving, regulatory frameworks demonstrate that responsible governance and scientific advancement are not contradictory (Bloomfield et al., 2026). Insights from agrifood biotechnology biosafety indicate that a completely unified regulatory body may not be necessary. In this regard, the practices of countries and regions with well-established regulatory systems, such as Canada, the United States, and Mercosur (Hoffman, 2021; 2022; Rocha-Salavarrieta, 2022; Lubieniechi et al., 2025b; 2025a; Ludlow et al., 2025) demonstrate how domestic science-based frameworks can effectively support GEd governance. These approaches also reveal convergence toward similar principles for evaluating such products.

Emerging clinical evidence, including reported adverse outcomes of gene therapy, may also influence public perceptions by contributing to uncertainty and increasing ethical scrutiny. However, even in regulatory systems that function effectively, ethical concerns are reconfigured, as the flexibility required to keep pace with technological advances may conflict with the stability and legitimacy of the decisions. Frequent changes can generate perceptions of volatility or arbitrariness, thereby weakening public trust.

The challenge is not to eliminate uncertainty or ethical controversy, both of which are inherent to emerging technologies. Instead, it lies in developing governance mechanisms that preserve transparency, consistency, and clear criteria so that evidence-based and-evidence informed revisions strengthen rather than weaken the legitimacy of regulatory processes (Bryant and Baggott la Velle, 2019c). This is particularly relevant when distinguishing between therapeutic versus enhancement-oriented uses, as each raises different ethical concerns and may require differentiated regulatory approaches. Public responses, in turn, are shaped not only by risk assessments, but also by social and cultural values and intuitive reactions. Otherwise, technological capability may outpace society’s capacity to shape its legitimate direction.

## Footnotes

1 Many GEd applications are grouped under the term “New Breeding Techniques” (NBTs), a category introduced to encompass emerging technologies that harness modern biotechnology to generate genetic diversity. However, as a class, NBTs lack universally agreed-upon definitions. Techniques previously classified under this umbrella include genome editing, epigenetic modification, agroinfiltration, cis- and intragenesis, grafting with genetically modified material, reverse breeding, RNA interference, and gene drives. For the purposes of this study, the term “NBTs” is used interchangeably with “precision breeding,” “new plant breeding techniques,” or “new genomic techniques” (Rosado and Eriksson, 2022). Regardless of the term used to refer to this set of techniques, they all include GEd.

2 Product-based approach refers to regulatory frameworks in which oversight and regulatory requirements are determined by the characteristics of the final product, rather than by the process or technique used to obtain it (McHughen, 2016; Fernández Ríos et al., 2025a; Mewett et al., 2026).

3 For example, in Argentina, a novel combination of genetic material is considered to exist when a genome intervention results in a stable insertion and the following criteria are met: the genotype cannot be obtained through conventional breeding or arise spontaneously in nature, and the intervention results produces an expression product that is novel to the species’ gene pool (Secretaría de Agricultura, Ganadería y Pesca, Ministerio de Economía, 2026).

4 These challenges generate market risks for smaller-scale developers despite the reduction in entry costs offered by GEd, by reducing technical complexity and initial infrastructure requirements compared with other modern biotechnological techniques such as transgenesis (Bullock et al., 2021; Tachikawa and Matsuo, 2023).

5 “Normative convergence represents a process whereby the regulatory requirements across countries or regions become more similar or aligned over time as a result of the gradual adoption of internationally recognized technical guidance documents, standards and scientific principles, common or similar practices and procedures, or adoption of regulatory mechanisms that might be specific to a local legal context but that align with shared principles to achieve a common goal” (USFDA, 2021).

6 Unlike somatic cell interventions, germline modifications can be transmitted to future generations (Marchant, 2021).

7 Reported adverse outcomes of gene therapies, including insertional effects, immune-mediated reactions, and organ-specific toxicities (Qie et al., 2025; Kantor et al., 2026), as well as unintended effects such as off-target events (Ayanoğlu et al., 2020; Riveros Maidana et al., 2020), illustrate how emerging evidence informs about perceptions (John and Czechowicz, 2025; Rieske et al., 2026). Such events have been reported in specific clinical contexts involving patients with severe or rare conditions, as documented in regulatory and clinical studies (e.g., selected cases such as Skysona, Zolgensma, Elevidys, Strimvelis, Glybera).

## In memoriam

The authors dedicate this work to the memory of Marcos Antonio De Jesús Bogado Barboza, whose life inspired their commitment to dignity, responsibility, and respect for human values. Marcos’s influence resonates in every reflection on the relationship between biotechnology and society. His memory is woven into the questions that guided this study.
